# Curability of Tabes by Ehrlich’s Salvarsan and Neosalvarsan

**Published:** 1913-04

**Authors:** 


### ABSTRACTS.

Curability of Tabes by Ehrlich's Salvarsan and Neosalvarsan. Leredde, *Society of Medicine of Paris,* February 7, 1913. After discussing a large series of cases, the author reaches the following conclusions, no one dissenting, except as to the propriety of the word *cure.*. Tabes is not an entity but a syndrome and the termination of a syphilitic meningitis, extending to the roots and posterior bands of the spinal cord. The curability by mercury is certain in the sense of rendering a positive reaction negative. But, especially from the practical symptomatic side, salvarsan or, rather, neosalvarsan, should replace mercury, as its action is more rapid and more certain. The dosage should be 1 centigram of salvarsan or 1½ centigrams of neosalvarsan per kilo of body weight, three or four doses are given, checked by Wassermann reaction, at intervals of a month. In quite a number of cases, larger doses were given after the initial dose. He considers Herxheimer's reaction as an indication of a normal response to treatment and declares that unfavorable results have always been due to too large dose, faulty technic or too short intervals of treatment.

